# Effect of Tumor Necrosis Factor Family Member LIGHT (TNFSF14) on the Activation of Basophils and Eosinophils Interacting with Bronchial Epithelial Cells

**DOI:** 10.1155/2014/136463

**Published:** 2014-03-25

**Authors:** Huai Na Qiu, Chun Kwok Wong, Jie Dong, Christopher Wai-Kei Lam, Zhe Cai

**Affiliations:** ^1^Department of Chemical Pathology, The Chinese University of Hong Kong, Prince of Wales Hospital, Shatin, New Territories, Hong Kong; ^2^Institute of Chinese Medicine and State Key Laboratory of Phytochemistry and Plant Resources in West China, The Chinese University of Hong Kong, Hong Kong; ^3^Shenzhen Research Institute, The Chinese University of Hong Kong, Shenzhen, China; ^4^State Key Laboratory of Quality Research in Chinese Medicine, Macau Institute for Applied Research in Medicine and Health, Macau University of Science and Technology, Taipa, Macau

## Abstract

Allergic asthma can cause airway structural remodeling, involving the accumulation of extracellular matrix and thickening of smooth muscle. Tumor necrosis factor (TNF) family ligand LIGHT (TNFSF14) is a cytokine that binds herpesvirus entry mediator (HVEM)/TNFRSF14 and lymphotoxin **β** receptor (LT**β**R). LIGHT induces asthmatic cytokine IL-13 and fibrogenic cytokine transforming growth factor-**β** release from allergic asthma-related eosinophils expressing HVEM and alveolar macrophages expressing LT**β**R, respectively, thereby playing crucial roles in asthmatic airway remodeling. In this study, we investigated the effects of LIGHT on the coculture of human basophils/eosinophils and bronchial epithelial BEAS-2B cells. The expression of adhesion molecules, cytokines/chemokines, and matrix metalloproteinases (MMP) was measured by flow cytometry, multiplex, assay or ELISA. Results showed that LIGHT could significantly promote intercellular adhesion, cell surface expression of intercellular adhesion molecule-1, release of airway remodeling-related IL-6, CXCL8, and MMP-9 from BEAS-2B cells upon interaction with basophils/eosinophils, probably via the intercellular interaction, cell surface receptors HVEM and LT**β**R on BEAS-2B cells, and extracellular signal-regulated kinase, p38 mitogen activated protein kinase, and NF-**κ**B signaling pathways. The above results, therefore, enhance our understanding of the immunopathological roles of LIGHT in allergic asthma and shed light on the potential therapeutic targets for airway remodeling.

## 1. Introduction

Allergic asthma can result in airway remodeling and pulmonary fibrosis [1]. Airway remodeling is characterized by the accumulation of extracellular matrix (ECM), such as collagen, and thickening of smooth muscle. Fibrogenic cytokine transforming growth factor (TGF-*β*) and asthma-related IL-13 are crucial cytokines for synergistic airway remodeling [1]. Matrix metallopeptidase 9 (MMP-9), one of the extracellular proteases family members, mediates the degradation of the extracellular matrix during tissue remodeling [2]. 

Granulocyte basophils have been demonstrated to bind IgE and perform essential roles of Th2 cytokine-dependent immunity and allergic inflammation [3]. Basophils are rarely found in normal tissues. However, their number increases markedly at allergic inflammatory sites in the airways of asthmatic patients, especially during asthma exacerbation in response to allergen inhalation [4–6]. The granulocyte eosinophil is another principal effector cell of allergic inflammation [7]. Allergic asthma is characterized by the accumulation and infiltration of eosinophils in tissues mediated by the specific eosinophil chemokine eotaxin and vascular cell adhesion molecule (VCAM)-1 and intercellular adhesion molecule (ICAM)-1 on epithelial cells, with subsequent release of granular toxic proteins such as eosinophilic cationic protein from eoisnophils [7].

LIGHT (lymphotoxin-related inducible ligand that competes for glycoprotein D binding to herpesvirus entry mediator on T cells), also known as tumor necrosis factor superfamily (TNFSF)14/CD258, is one of the TNF family members. It is a homotrimer on the surface of several immune cells such as activated T and B cells. Many members including TNF-*α*, CD40 ligand (CD40L), Fas ligand (FasL), TNF-related activation-induced cytokine (TRANCE), and LIGHT can be cleaved from cell surfaces, and their soluble forms have been reported to be involved in various physiological processes with broad biological functions [8–11]. Since LIGHT is a membrane-expressed protein related to the membrane form of lymphotoxin (LT)*αβ* [12], it binds the herpesvirus entry mediator (HVEM; TNFRSF14) and is also a shared ligand with membrane lymphotoxin for LT*β*R [12, 13]. LIGHT can optimize inflammatory cytokine IL-12 production by dendritic cells and Th1 cells [14]. It is expressed on lung inflammatory CD45+leukocytes after the allergen house dust mite challenge [15]. LIGHT directly induces airway remodeling which is dependent on the induction of the fibrogenic cytokine transforming growth factor (TGF)-*β*. In mouse models of chronic asthma, pharmacological inhibition of LIGHT using a fusion protein between the IgG Fc domain and LT*β*R can reduce lung fibrosis, smooth muscle/epithelial hyperplasia, and airway hyperresponsiveness via the suppression of the production of lung TGF-*β* and IL-13, which are key cytokines in airway remodeling in humans [15]. On the other hand, exogenous administration of LIGHT to the airways induces fibrosis and smooth muscle hyperplasia. LIGHT-deficient mice exhibit impairment in fibrosis and smooth muscle accumulation [15]. In line with this, sputum LIGHT levels in asthmatic patients were found to correlate with decreased lung function [16]. Apart from LIGHT, anti-human B- and T-lymphocyte attenuator (BTLA), an inhibitory receptor on T lymphocytes with similar T-cell inhibitory functions to cytotoxic T lymphocyte-associated antigen 4 (CTLA-4) and programmed death 1 (PD-1) [17], is also the ligand of HVEM [18, 19]. Although BTLA-HVEM complexes have been shown to negatively regulate T-cell immune responses [18], the expression of BTLA on asthma-related basophils, eosinophils, and bronchial epithelial cells have not been investigated.

Recent mechanistic studies have shown that LIGHT can induce IL-13 and TGF-*β* release from esoinophils and alveolar macrophages, respectively [15, 20]. Eosinophils express HVEM but not LT*β*R [15], while LIGHT can induce MMP-9 release from macrophages via LT*β*R [21]. We have recently demonstrated the crucial roles of the interaction of basophils and eosinophils with bronchial epithelial cells in allergic asthma [22, 23]. However, the precise role played by the elevated LIGHT in airway hyperresponsiveness is still unresolved, and the intracellular mechanisms by which LIGHT can activate bronchial epithelial cells interacting with basophils and eosinophils to release airway remodeling related molecules are not certain. Since we hypothesize that LIGHT may play an immunological role in airway remodeling through the activation of the intercellular interaction between the granulocyte and airway epithelium, the aim of the present study was to investigate the effects of LIGHT on bronchial epithelial cells interacting with basophils/eosinophils and the underlying intracellular mechanisms.

## 2. Materials and Methods

### 2.1. Reagents

The recombinant human LIGHT/TNFSF14 was purchased from R&D Systems (Minneapolis, MN, USA). I*κ*B*α* phosphorylation inhibitor BAY11-7082, p38 MAPK inhibitor SB203580, c-Jun N-terminal protein kinase (JNK) inhibitor SP600125, extracellular signal-regulated kinase (ERK) inhibitor U0126, and PI3K inhibitor LY294002 were purchased from Calbiochem Corporation (San Diego, CA, USA). BAY11-7082, SB203580, SP600125, U0126, and LY294002 were dissolved in 0.1% (v/v) dimethylsulphoxide (DMSO).

### 2.2. Purification of Human Peripheral Blood Basophils and Eosinophils from Buffy Coat and Cell Culture

Purification of human basophils and eosinophils was performed according to our previous publications [22–24]. Fresh human buffy coat obtained from healthy volunteers of the Hong Kong Red Cross Blood Transfusion Service was diluted with PBS and centrifuged using Ficoll-Paque Plus solution (GE Healthcare Corp., Piscataway, NJ, USA) and isotonic Percoll solution (density 1.082 g/mL; GE Healthcare) for the purification of basophils and eosinophils, respectively. Basophil-rich peripheral blood mononuclear cell (PBMC) fraction or eosinophil-rich granulocyte fraction was collected and washed twice with cold PBS containing 2% fetal bovine serum (FBS) (Invitrogen Corp., Carlsbad, CA, USA). Basophils and eosinophils were purified by negative selection using basophil isolation kit and anti-CD16 magnetic beads (Miltenyi Biotec, Bergisch Gladbach, Germany), respectively, using an LS+ column (Miltenyi) within a magnetic field. With this preparation, the drop-through fraction contained purified basophils or eosinophils with a purity of at least 99% as assessed by Giemsa staining solution (Sigma-Aldrich Corp., St. Louis, MO, USA) together with specific basophil cell surface marker CD203c staining [22] or Hemacolor rapid blood smear stain (E Merck Diagnostica, Darmstadt, Germany) [23], respectively. The isolated basophils/eosinophils were cultured in RPMI1640 medium (Invitrogen) supplemented with 10% FBS (Invitrogen). The above protocol using human basophils/eosinophils purified from human buffy coat was approved by the Clinical Research Ethics Committee of The Chinese University of Hong Kong-New Territories East Cluster Hospitals with written consent from all healthy volunteers of Hong Kong Red Cross Blood Transfusion Service in accordance with the Declaration of Helsinki.

### 2.3. Coculture of Basophils/Eosinophils and Bronchial Epithelial Cells

The human bronchial epithelial cell line (BEAS-2B) was obtained from the American Type Culture Collection (ATCC, Manassas, VA, USA). This cell line has been transformed by adenovirus 12-SV40 virus hybrid (Ad12SV40) and used widely as an* in vitro *bronchial epithelial cell model [24]. BEAS-2B cells were grown in Dulbecco's modified Eagle's medium nutrient mixture F12 (Invitrogen) with 10% FBS at 37°C in a humidified 5% CO_2_ atmosphere until confluence to cell monolayer. In coculture, the medium of BEAS-2B cells was replaced with RPMI-1640 medium containing 10% FBS (Invitrogen) with or without basophils/eosinophils. For inhibition experiments, basophils/eosinophils and BEAS-2B cells were pretreated with signaling molecule inhibitors for 1 h before coculture and treatment by LIGHT.

### 2.4. Coculture of Basophils/Eosinophils and BEAS-2B Cells in the Presence of Transwell Inserts

To prevent direct interaction between basophils/eosinophils and BEAS-2B cells in the coculture, transwell inserts (pore size: 0.4 mM) (BD Biosciences Corp., San Jose, CA, USA) were used to separate these two cells into two compartments. Confluent BEAS-2B cells and basophils/eosinophils were cultured together in the presence of transwell inserts, in which basophils/eosinophils and BEAS-2B cells were placed in the upper and lower compartment, respectively [23].

### 2.5. Quantification of Cytokines, Chemokines, and Growth Factors Using Multiplex Immunoassay

Concentrations of cytokines IL-5, IL-6, IL-9, IL-13, epidermal growth factor (EGF), vascular endothelial growth factor (VEGF), TGF-*β*, and chemokine CXCL8 in the culture supernatants were measured using the human Milliplex MAP kit assay reagent (Merck Millipore Corp., Billerica, MA, USA) with Bio-Plex 200 suspension array system (Bio-Rad Laboratories, Inc., Hercules, CA, USA).

### 2.6. Quantification of Human MMP-9 and Periostin

Concentration of human MMP-9 in the culture supernatants was measured using the Milliplex human MMP magnetic panel assay reagent (Merck Millipore) with Bio-Plex 200 suspension array system (Bio-Rad). Human periostin was measured using ELISA reagent (RayBiotech Inc., GA, USA).

### 2.7. Adhesion Assay

Coculture of BEAS-2B cells (1 × 10^5^ cells) and basophils/eosinophils (3 × 10^5^ cells) or BEAS-2B cells alone (3 × 10^5^ cells) were maintained in a 24-well plate with transwell inserts (pore size: 0.4 mM) (BD Biosciences) and stimulated with LIGHT (0–100 ng/mL) for 24 h. The basophils/eosinophils were removed and another batch of freshly isolated basophils/eosinophils (3 × 10^5^ cells) was then added into the adherent BEAS-2B cells for the adhesion analysis. Cells were cultured in RPMI1640 medium containing 10% FBS and incubated at 37°C in a humidified 5% CO_2_ atmosphere for 1 h and digested with trypsin and resuspended in sheath fluid. Basophils/eosinophils and BEAS-2B cells were analyzed separately based on the expression of specific basophilic cell surface marker CD203c in histograms and distinct forward light scatters (FSC) together with side light scatters (SSC) of eosinophils in dot plots using flow cytometry (FACSCalibur flow cytometer, BD Biosciences). The ratio of the measured number of adherent eosinophils/basophils onto BEAS-2B cells was calculated [22–24].

### 2.8. Immunofluorescence Staining and Flow Cytometric Analysis

To determine the expression of HVEM, LT*β*R, and intercellular adhesion molecule (ICAM)-1 on the cell surface, nonadherent basophils/eosinophils were washed and resuspended with cold PBS. Adherent bronchial epithelial cells were harvested using cell dissociation solution. After blocking with 2% human pooled serum for 20 min at 4°C and washing with PBS supplemented with 0.5% bovine serum albumin, cells were incubated with phycoerythrin (PE)-conjugated mouse anti-human HVEM antibody, PE-conjugated mouse anti-human LT*β*R antibody, APC-conjugated mouse anti-human BTLA/CD272 antibody (BioLegend, Inc., San Diego, CA, USA), PE-conjugated mouse IgG1 isotype, fluorescein isothiocyanate (FITC)-conjugated mouse anti-human ICAM-1 antibody, or FITC-conjugated mouse IgG2a, *κ* isotype (BioLegend) for 30 min at 4°C in the dark. After washing, cells were subjected to flow cytometric analysis [22].

To determine the intracellular expression of phosphorylated signaling molecules, cells were fixed with prewarmed 4% paraformaldehyde for 10 min at 37°C. After centrifugation, cells were permeabilized in ice-cold BD Phosflow Perm Buffer for 30 min and then stained with mouse anti-human phosphorylated (p) p38 MAPK, pERK1/2, pI*κ*B*α*, or mouse IgG1 antibodies (BD Pharmingen Corp., San Diego, CA, USA) for 60 min followed by FITC-conjugated goat anti-mouse secondary antibody (Life Technologies, Carlsbad, CA, USA) for another 45 min at 4°C in the dark. Cells were then washed, resuspended, and subjected to flow cytometric analysis [22].

Expression of surface molecules and intracellular phosphorylated signaling molecules of 5,000 viable cells was analyzed using flow cytometry (BD FACSCalibur flow cytometer) and presented as mean fluorescence intensity (MFI). For the differential analysis of intracellular MAPK and nuclear factor (NF)-*κ*B activity of BEAS-2B cells, nonadherent basophils/eosinophils were separated from the adherent BEAS-2B cells by washing with PBS after different treatments. Adherent BEAS-2B cells were then harvested using cell dissociation solution for the flow cytometric analysis of intracellular signaling molecules (Sigma Aldrich Corp., MO, USA). Basophils/eosinophils and BEAS-2B cells were analyzed separately based on the expression of specific basophilic cell surface marker CD203c in histograms and distinct forward light scatters (FSC) together with side light scatters (SSC) of eosinophils in dot plots using flow cytometry (FACSCalibur flow cytometer, BD Biosciences) [22–24].

### 2.9. Statistical Analysis

The statistical significance difference was determined by one-way analysis of variance (ANOVA) or unpaired *t*-test. Data were expressed as mean plus standard error of the mean (SEM) from three independent experiments. Any differences with *P* value <0.05 were considered significant. When ANOVA indicated a significant difference, Bonferroni's* post hoc *test was then used to assess the difference between groups. All analyses were performed using SPSS statistical software for Windows (version 16.0; SPSS Inc., Chicago, IL, USA).

## 3. Results

### 3.1. Cell Surface Expression of HVEM, LT*β*R, and BTLA

**Figure 1:**
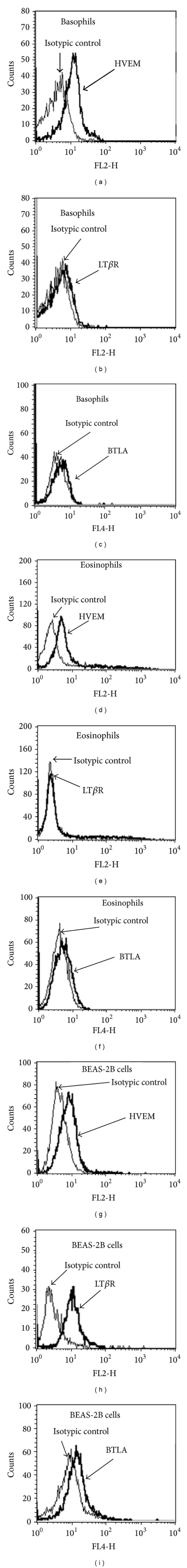
Protein expression of HVEM, LT*β*R, and BTLA on purified human basophils, purified human eosinophils, and human bronchial epithelial BEAS-2B cells. Representative histograms of the cell surface expression of HVEM, LT*β*R, and BTLA on (a, b, c) basophils (d, e, f), eosinophils, and (g, h, i) BEAS-2B cells determined with gating by respective side scatter and forward scatter using flow cytometry were obtained from triplicate experiments with essentially identical results.

As shown in Figures 1(a), 1(d), 1(g), 1(h), and 1(i), the proteins HVEM, LT*β*R, and BTLA were constitutively expressed on the cell surface of bronchial epithelial BEAS-2B cells but only HVEM was observed to be expressed on basophils and eosinophils. Results were similar to a previous report [15] that eosinophils expressed cell surface HVEM but not LT*β*R (Figures 1(d) and 1(e)). Consistent with a previous publication, a slight decrease in HVEM expression on these cells was detected after LIGHT stimulation [25] (data not shown).

### 3.2. Effects of LIGHT on the Expression of ICAM-1 on BEAS-2B Cells and Eosinophils

**Figure 2:**
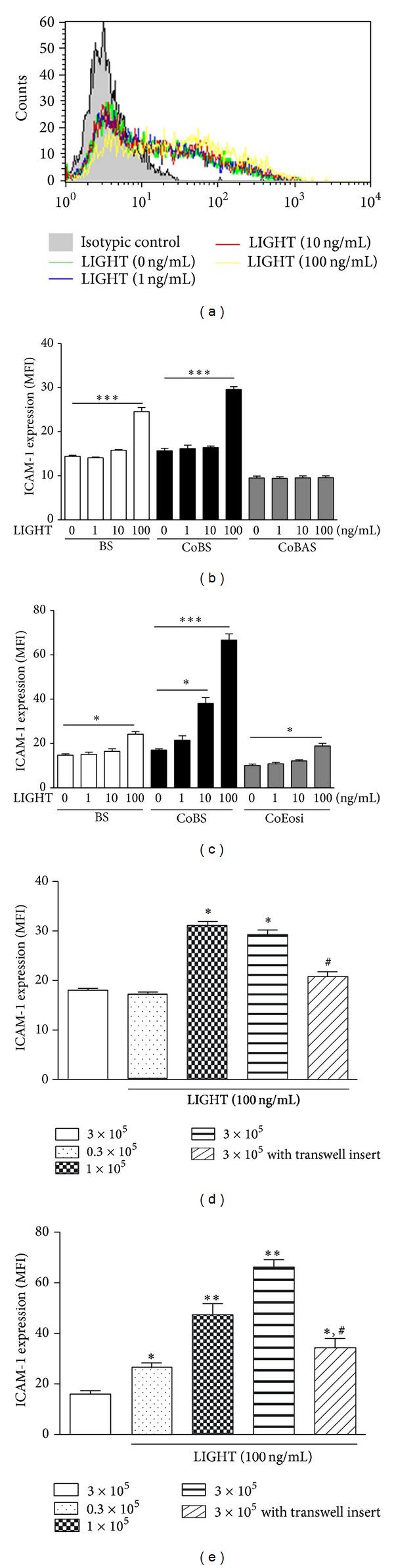
Effect of LIGHT on the cell surface expression of ICAM-1 on BEAS-2B cells or basophils/eosinophils. Expressions of ICAM-1 on BEAS-2B cells alone or in the coculture, and basophils/eosinophils in the coculture with or without LIGHT stimulation are presented with representative bar charts. (a) Representative histogram of the cell surface expression of ICAM-1 on BEAS-2B cells (1 × 10^5^ cells) treated with different concentration of LIGHT (0–100 ng/mL) for 24 h is shown. (b) Basophils or (c) eosinophils (3 × 10^5^ cells) and confluent BEAS-2B cells (1 × 10^5^ cells) were cultured either together or separately with or without LIGHT (1–100 ng/mL) for 24 h. Surface expressions of ICAM-1 on 5,000 BEAS-2B cells, basophils, or eosinophils are expressed as the mean plus SEM of MFI of three independent experiments with three blood samples. *P < 0.05, ***P < 0.001. (d) Basophils or (e) eosinophils (0.3 × 10^5^–3 × 10^5^ cells) and confluent BEAS-2B cells (1 × 10^5^ cells) were cultured together with or without LIGHT (100 ng/mL) and transwell (pore size 0.4 *μ*m) for 24 h. Surface expressions of ICAM-1 on 5,000 BEAS-2B cells, basophils, or eosinophils are expressed as the mean plus SEM of MFI of three independent experiments with three blood samples. *P < 0.05; **P < 0.01 when compared with the cocultured BEAS-2B cells and eosinophils without stimulation with LIGHT (empty bar). ^#^ *P* < 0.05 when compared with the corresponding group without transwell inserts. BS: BEAS-2B cells alone; CoBS: BEAS-2B cells in the coculture; CoBAS: basophils in the coculture; CoEosi: eosinophils in the coculture.

Since we observed that plasma LIGHT concentration of asthmatic patients using ELISA could be up to 100 pg/mL, the local inflammatory concentration could be 10–1000 fold higher than the circulating levels. In order to mimic the inflammatory condition, we chose 1–100 ng/mL concentration for the following* in vitro* studies that is also comparable to that adopted in a previous publication [26].  Figure 2(a) shows that LIGHT (100 ng/mL) could upregulate the cell surface expression of ICAM-1 on BEAS-2B cells alone. As shown in Figure 2(b), the cell surface expression of ICAM-1 on BEAS-2B cells alone was significantly enhanced upon stimulation by LIGHT at high concentration (100 ng/mL) (*P* < 0.05) but not with low concentration (1 or 10 ng/mL). Upon interaction with basophils, ICAM-1 level on BEAS-2B cells was also upregulated by LIGHT at 100 ng/mL only (Figure 2(b)). However, LIGHT (up to 100 ng/mL) did not show any significant effect on the expression of ICAM-1 on basophils in the coculture with BEAS-2B cells (Figure 2(b), all *P* > 0.05). In the coculture of eosinophils and BEAS-2B cells (Figure 2(c)), LIGHT could significantly induce the expression of ICAM-1 on BEAS-2B cells (LIGHT, 10 and 100 ng/mL) and eosinophils (LIGHT, 100 ng/mL) (all *P* < 0.05). As shown in Figures 2(d) and 2(e), the increased cell number of basophils or eosinophils (0.3 × 10^5^–3 × 10^5^ cells) could enhance the expression of ICAM-1 on BEAS-2B cells in coculture. Moreover, the transwell insert could significantly downregulate the ICAM-1 expression on BEAS-2B cells in coculture (all *P* < 0.05).

We did not observe any significant changes in cell surface expression of ICAM-1 on basophils or eosinophils alone upon treatment with LIGHT up to 100 ng/mL (*P* > 0.05) (data not shown).

### 3.3. Adhesion of Basophils/Eosinophils onto BEAS-2B Cells

**Figure 3:**
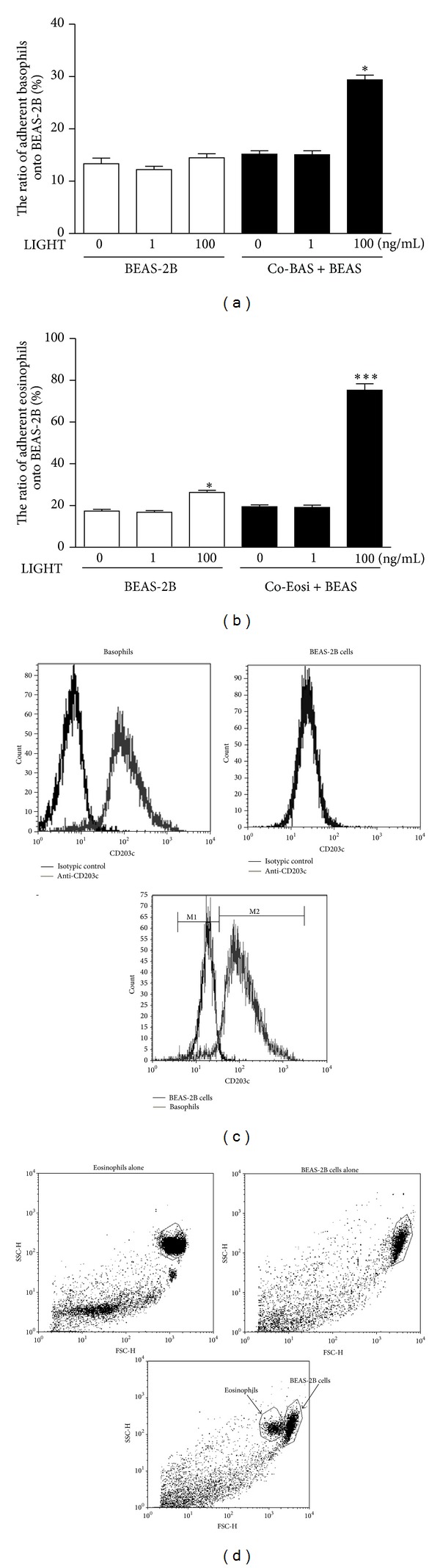
Adhesion of basophils or eosinophils onto BEAS-2B cells. Coculture of BEAS-2B cells (1 × 10^5^ cells) and basophils/eosinophils (3 × 10^5^ cells) using transwell inserts or BEAS-2B cells alone were stimulated by LIGHT (0–100 ng/mL) for 24 h. Basophils/eosinophils were removed and freshly isolated basophils/eosinophils (3 × 10^5^ cells) were added into the corresponding well containing the adherent BEAS-2B cells (1 × 10^5^ cells) for 1 h incubation. The ratio of (a) basophils or (b) eosinophils adherent onto BEAS-2B was analysed using flow cytometry as described in Section 2. Results are expressed as the mean plus SEM of three independent experiments with three blood samples. *P < 0.05, ***P < 0.001. BEAS-2B: BEAS-2B cells alone were treated with LIGHT prior to adhesion analysis; Co-BAS + BEAS-2B: Coculture of BEAS-2B cells and basophils were treated with LIGHT before BEAS-2B cells were used for adhesion analysis; Co-Eosi + BEAS-2B: Coculture of BEAS-2B cells and eosinophils were treated with LIGHT before BEAS-2B cells were used for adhesion analysis. In the above adhesion assay, basophils/eosinophils and BEAS-2B cells were analyzed separately using flow cytometry. (c) Representative histograms of the flow cytometric analysis of the number of adherent basophils in coculture gated by the specific cell surface basophilic marker CD203c were shown. (d) Representative dot plots of the flow cytometric analysis of the number of adherent eosinophils in coculture gated by the SSC and FSC were shown.

To further address the LIGHT-induced activation in cocultured BEAS-2B and basophils/eosinophils, we estimated the ability of activated BEAS-2B in adhesion with basophils/eosinophils (Figure 3). An increased number of adherent basophils/eosinophils could only be observed in coculture upon treatment by 100 ng/mL LIGHT (Figures 3(a) and 3(b), both *P* < 0.05).  Figure 3(b) shows that there was a moderate upregulation of eosinophils adhesion onto BEAS-2B cells when BEAS-2B cells alone were stimulated with LIGHT (100 ng/mL). The above results therefore were in concordance with the upregulated expression of adhesion molecule on BEAS-2B cocultured with basophils/eosinophils in the presence of LIGHT (Figure 2). Figures 3(c) and 3(d) showed that basophils/eosinophils and BEAS-2B cells could be analyzed separately based on the expression of specific basophilic cell surface marker CD203c on basophils (Figure 3(c)) and distinct forward light scatters (FSC) together with side light scatters (SSC) of eosinophils in dot plots (Figure 3(d)) using flow cytometry.

### 3.4. Induction of Cytokines and Chemokines upon the Interaction of Basophils/Eosinophils and BEAS-2B Cells Stimulated by LIGHT

**Figure 4:**
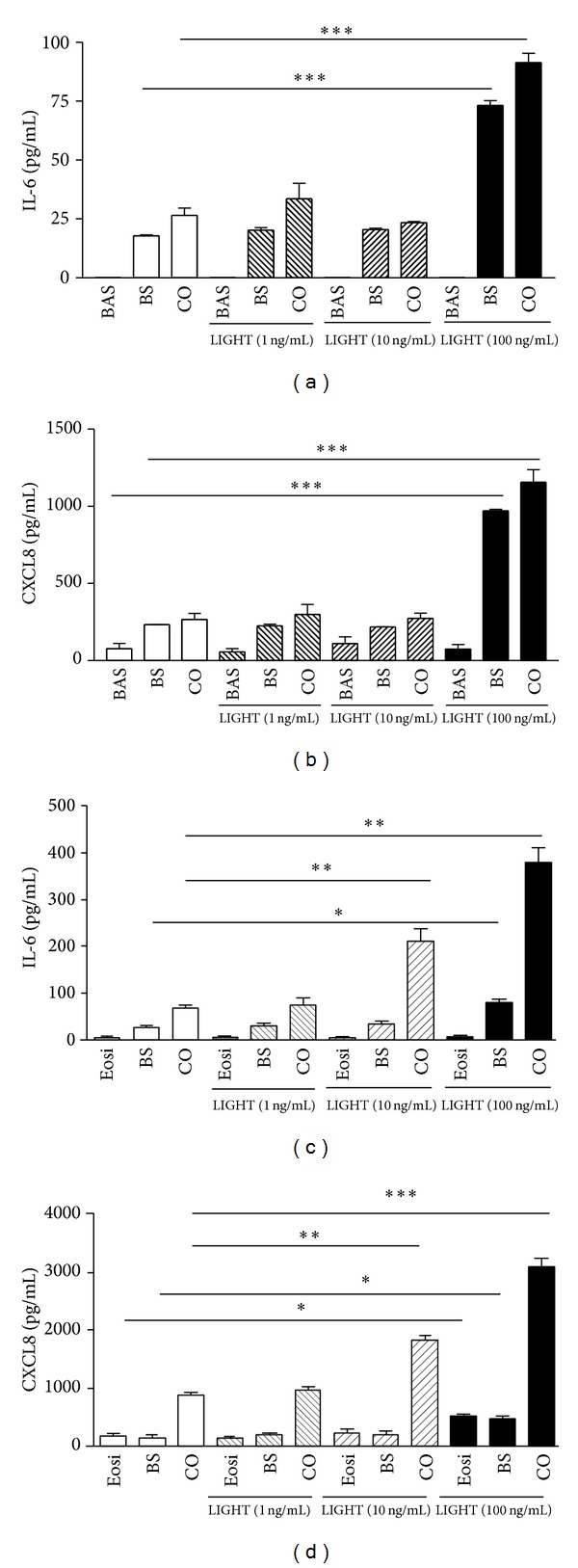
Effects of LIGHT on the release of IL-6 and CXCL8 from the coculture of basophils/eosinophils and BEAS-2B cells. Confluent BEAS-2B cells (1 × 10^5^ cells) and (a, b) basophils/(c, d) eosinophils (3 × 10^5^ cells) were cultured either together or separately with or without LIGHT for 24 h. Release of IL-6 and CXCL8 in culture supernatants was determined by Milliplex human cytokine/chemokine magnetic panel assay. Results are expressed as the mean plus SEM of three independent experiments with three blood samples. *P < 0.05, **P < 0.01, ***P < 0.001. BAS: basophils; Eosi: eosinophils; BS: BEAS-2B cells; CO: coculture.

The Milliplex human cytokine/chemokine magnetic panel assay was used to measure the airway remodeling-related cytokines and chemokines, including IL-5, IL-6, IL-9, IL-13, EGF, TGF-*β*, VEGF, and CXCL8. As shown in Figures 4(a) and 4(b), LIGHT (100 ng/mL) could significantly induce the release of cytokine IL-6 and chemokine CXCL8 from BEAS-2B cells. Upon coculture with basophils, the induction of IL-6 and CXCL8 by LIGHT (100 ng/mL) was found to be significantly higher than those of BEAS-2B cells alone (both *P* < 0.05). However, LIGHT did not show any significant effect on the release of IL-6 or CXCL8 from basophils alone, even at high concentration (100 ng/mL, Figures 4(a) and 4(b)).  Figure 4(d) shows that LIGHT (100 ng/mL) could significantly promote the release of CXCL8 from eosinophils alone. LIGHT (10 ng/mL) could further induce the release of IL-6 and CXCL8 from coculture of eosinophils and BEAS-2B cells compared to eosinophils or BEAS-2B cells alone (all *P* < 0.05, Figures 4(c) and 4(d)). In addition, the coculture of BEAS-2B cells with eosinophils, together with LIGHT stimulation (100 ng/mL), exhibited a synergistic effect on IL-6 and CXCL8 production (all *P* < 0.05, Figures 4(c) and 4(d)). The levels of IL-5, IL-9, IL-13, EGF, VEGF, and periostin were all undetectable using the same experimental conditions (data not shown). Moreover, there was induction of IL-6 and CXCL8 production from the coculture BEAS-2B cells and eosinophils without LIGHT stimulation (Figure 4). We also found no significant induction of TGF-*β* in the coculture of BEAS-2B cells and basophils/eosinophils with or without LIGHT stimulation (all *P* > 0.05, data not shown).

### 3.5. Induction of MMP-9 upon the Interaction of Basophils and BEAS-2B Cells Stimulated by LIGHT

**Figure 5:**
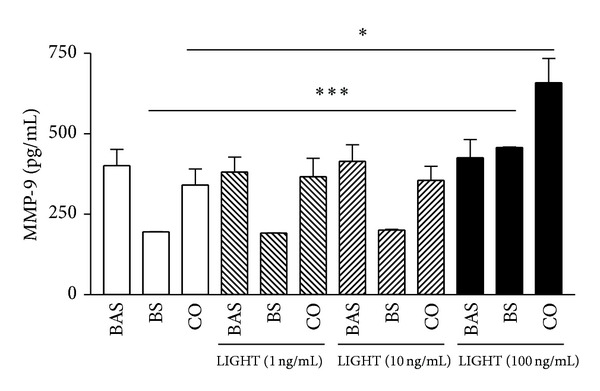
Effects of LIGHT on the release of MMP-9 in the coculture of basophils and BEAS-2B cells. Confluent BEAS-2B cells (1 × 10^5^ cells) and basophils (3 × 10^5^ cells) were cultured either together or separately with LIGHT (0–100 ng/mL) for 24 h. Release of MMP-9 in culture supernatants was measured by Milliplex human MMP panel assay. Results are expressed as the mean plus SEM of three independent experiments with three blood samples. *P < 0.05, ***P < 0.001. BAS: basophils; BS: BEAS-2B cells; CO: coculture of basophils and BEAS-2B cells.

As shown in Figure 5, LIGHT (100 ng/mL) could significantly induce the release of MMP-9 from BEAS-2B cells (*P* < 0.001). Upon coculture with basophils, the induction of MMP-9 by LIGHT (100 ng/mL) was significantly higher than control without LIGHT treatment (*P* < 0.05). However, LIGHT did not show any significant effect on the release of MMP-9 from basophils alone, even at high concentration (100 ng/mL, *P* > 0.05). There was no significant induction of MMP-9 in eosinophils alone or the coculture of eosinophils with BEAS-2B cells with or without LIGHT stimulation (data not shown).

### 3.6. Signaling Pathways Involved in the Interaction of Basophils/Eosinophils and BEAS-2B Cells upon LIGHT Stimulation

**Figure 6:**
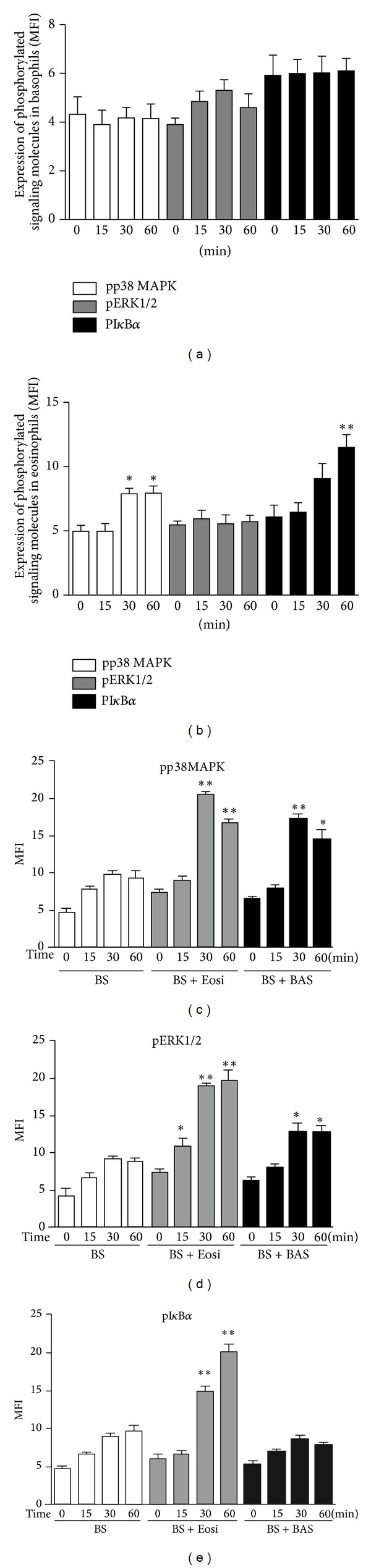
Phosphorylation of p38 MAPK, ERK1/2, and I*κ*B*α* in BEAS-2B cells upon the coculture of basophils/eosinophils and BEAS-2B cells with the stimulation of LIGHT. BEAS-2B cells (1 × 10^5^ cells) and basophils/eosinophils (3 × 10^5^ cells) were cultured either together or separately with or without LIGHT stimulation (100 ng/mL) for different time points (0, 15, 30, and 60 min). The intracellular expression of phosphorylated (p) p38 MAPK, pERK1/2, and pI*κ*B*α* in (a) permeabilized basophils and (b) eosinophils alone without coculture, and (c) pp38 MAPK, (d) pERK1/2, and (e) pI*κ*B*α* of permeabilized BEAS-2B cells in with or without coculture with basophils/eosinophils were measured by intracellular immunofluorescence staining using flow cytometry. Results are shown in MFI and expressed as the arithmetic mean plus SEM of three independent experiments with three blood samples in bar charts. *P < 0.05, **P < 0.01 when compared with coculture control group. BAS: basophils; Eosi: eosinophils; BS: BEAS-2B cells.

As shown in Figures 6(a) and 6(b), LIGHT (100 ng/mL) could significantly activate ERK and NF-*κ*B in eosinophils (all *P* < 0.05) but not in basophils (all *P* > 0.05). Figures 6(c), 6(d), and 6(e) show that with the treatment of LIGHT (100 ng/mL), phosphorylation of p38 MAPK, ERK1/2, and I*κ*B*α* was significantly enhanced in BEAS-2B cells upon their coculture with eosinophils at 30 and 60 min, and ERK1/2 was significantly phosphorylated even at 15 min. The p38 MAPK and ERK1/2 were significantly phosphorylated in BEAS-2B cells of the coculture of basophils and BEAS-2B cells at 30 and 60 min upon LIGHT (100 ng/mL) stimulation (Figures 6(c) and 6(d)).

### 3.7. Effects of Signaling Inhibitors on LIGHT-Induced Adhesion Molecule and Cytokines/Chemokines

**Figure 7:**
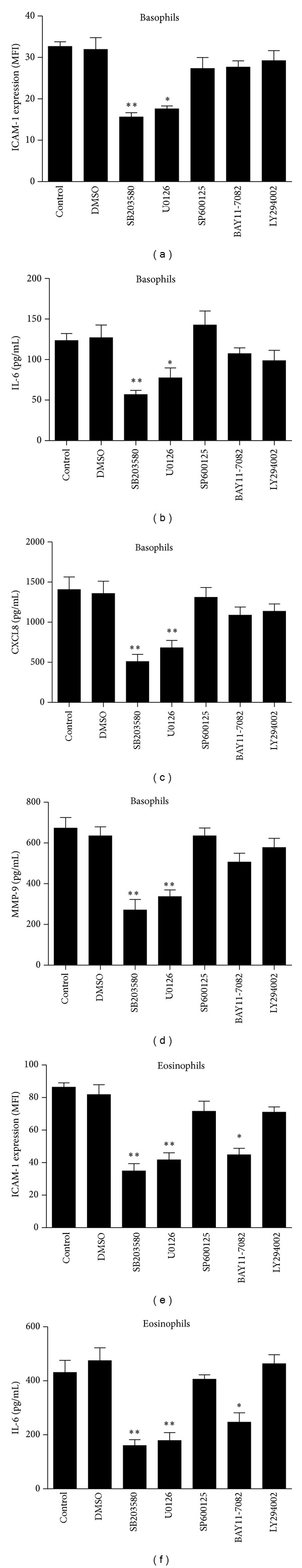
Effects of signaling molecule inhibitors on the cell surface expression of ICAM-1 and induction of cytokines/chemokines from coculture of BEAS-2B cells and basophils/eosinophils in the presence of LIGHT (100 ng/mL). Cocultures of (a, b, c, d) basophils or (e, f) eosinophils (3 × 10^5^ cells) and confluent BEAS-2B cells (1 × 10^5^ cells) were pretreated with BAY11-7082 (1 *μ*M), SP600125 (3 *μ*M), SB203580 (7.5 *μ*M), U0126 (10 *μ*M), or LY294002 (10 *μ*M) for 1 h, followed by incubation with or without LIGHT (100 *μ*g/mL) in the presence of inhibitors for a further 24 h. Cell surface expression of ICAM-1 on 5,000 cells was analyzed by flow cytometry as MFI. Results are expressed as mean plus SEM of three independent experiments with three blood samples in bar charts. Release of cytokines/chemokines and MMP-9 in culture supernatant was measured by Milliplex assay. Results are expressed as mean plus SEM. DMSO (0.1%) was used as the vehicle control. *P < 0.05, **P < 0.01 when compared with vehicle or negative control.

The p38 MAPK inhibitor SB203580 (7.5 *μ*M) and ERK inhibitor U0126 (10 *μ*M) could significantly suppress the LIGHT-induced expression of ICAM-1 on BEAS-2B cells in their coculture with basophils (Figure 7(a)) and the release of IL-6, CXCL8, and MMP-9 from the coculture (Figures 7(b), 7(c), and 7(d)). The p38 MAPK inhibitor SB203580 (7.5 *μ*M), ERK inhibitor U0126 (10 *μ*M), and NF-*κ*B inhibitor BAY11-7082 (1 *μ*M) could significantly suppress the LIGHT-induced expression of ICAM-1 on BEAS-2B cells (Figure 7(e)) and the production of IL-6 (Figure 7(f)) from the coculture of eosinophils and BEAS-2B cells.

## 4. Discussion

Structural remodeling of the airway involves the accumulation of extracellular matrix proteins and thickening of smooth muscle [1]. We found that allergic asthma-related basophils and eosinophils constitutively expressed HVEM (Figure 1), a ligand of airway remodeling cytokine LIGHT. Bronchial epithelial BEAS-2B cells also expressed HVEM, LT*β*R, and another HVEM ligand, BTLA [17]. Therefore, LIGHT can play immunomodulatory roles for the activation of basophils/eopsinophils interacting with bronchial epithelial cells in airway remodeling. The present* in vitro* study has shown that the TNF family member LIGHT could significantly promote the cell surface expression of adhesion molecule ICAM-1, the release of airway-remodeling cytokine IL-6, chemokine CXCL8, and extracellular protease MMP-9 from human bronchial epithelial cells upon the interaction with asthma-related basophils or eosinophils. Since the increased cell number of basophils/eosinophils could enhance the expression of ICAM-1 on BEAS-2B cells, and the disruption of intercellular interaction using transwell inserts could significantly downregulate the ICAM-1 expression on BEAS-2B cells (Figures 2(d) and 2(e)), upregulated ICAM-1 expression on BEAS-2B cells was dependent on the direct interaction between basophils/eosinophils and BEAS-2B cells.

As shown in Figure 2(e), there was a remarkable increase in the ICAM-1 expression on BEAS-2B interacted with eosinophils after stimulated with LIGHT in the presence of the transwell insert, suggesting that the soluble factors were also associated with the regulation of ICAM-1. Since the direct interaction of ICAM-1 and CD18 has been shown to mediate eosinophil adhesion onto bronchial epithelial cells [27], the increased ability of BEAS-2B in adhesion with eosinophils could be partially explained by the upregulated ICAM-1 on BEAS-2B upon interaction with eosinophils with LIGHT stimulation. However, the elevated adhesion between BEAS-2B and basophils could also be observed, even the ICAM-1 expression was similar between (i) BEAS-2B coculturing with basophils using transwell inserts (Figure 2(d)) and (ii) BEAS-2B cells alone were stimulated by LIGHT (Figure 2(b)), thereby suggesting that other factors in coculture, apart from the adhesion molecule ICAM-1, were partly contributing to the intercellular adhesion. Further study may be required to fully elucidate the biochemical mechanisms for the adhesion of basophils/eosinophils onto BEAS-2B cells.

IL-6 has been shown to enhance collagen synthesis, production of tissue inhibitors of metalloproteinases (TIMPs), and airway hyperresponsiveness [28–30]. Expression of IL-6 in fibroblasts is correlated with fibrosis [28]. By causing airway smooth muscle cell proliferation and migration, CXCL8 has been shown to be a potential chemokine for airway remodeling [31]. CXCL8 released from human bronchial epithelial cells upon leukotriene D4 stimulation is involved in epithelial-mediated asthmatic airway remodeling via the activation of EGF receptor [32]. MMP-9 is significantly increased in bronchoalveolar lavage fluid (BALF) and sputum from patients with allergic asthma [33, 34]. An elevated circulation level of MMP-9 is also found in patients suffering from asthma exacerbation [35]. In addition, allergen challenge in asthmatic patients induces MMP-9 expression in the airway [36]. MMP-9-deficient mice challenged with ovalbumin show less peribronchial fibrosis and total lung collagen compared to ovalbumin-challenged wild type [37]. These results indicated the involvement of MMP-9 in mediating allergen-induced airway remodeling [37]. Together, the induced IL-6, CXCL8, and MMP-9 suggested that LIGHT may play an important role in airway remolding via the activation of basophils and eosinophils interacting with bronchial epithelial cells.

There are three isoforms of TGF-*β* in the normal human lung, and TGF-*β*1 is associated with bronchial epithelial cells, smooth muscle cells, fibroblast-like cells, and the airway extracellular matrix (ECM) [38–41]. TGF-*β*1 level is increased in BALF of asthmatic patients [42], and asthmatic animal models also show increased levels of TGF-*β*1 in BALF and tissue [43, 44]. Mice treated with anti-TGF-*β* antibody significantly reduce the deposition of peribronchial ECM, proliferation of airway smooth muscle cell, and mucus production in lung [45]. TGF-*β* induces the expression of MMPs and TIMPs, both are major ECM regulators [46]. Deposition of ECM will result in fibrosis in patients with asthma [47]. TGF-*β*1 plays an important role in the regulation of airway remodeling. In the present study, BEAS-2B cells were found to release TGF-*β*1; however, the levels of TGF-*β*1 seemed not to be influenced by LIGHT or the interaction with basophils or eosinophils (data not shown). It has been shown that the matricellular protein periostin can interact with cell surface integrin molecules and can be involved in tissue development and remodeling [48, 49]. However, in the present study, the level of periostin is undetectable by ELISA (data not shown). Moreover, other airway remodeling-related cytokines and growth factors including IL-5, IL-9, IL-13, EGF, and VEGF could not be detected in the present study. The expression of these mediators should be further investigated in the future* in vivo *study using murine model to better elucidate the detailed effect of LIGHT on the interaction of basophils/eosinophils and bronchial epithelial cells in airway remodeling and inflammation.

Our previous study of airway inflammation has demonstrated that the induction of IL-6 and CCL2 upon the interaction of basophils and bronchial epithelial cells under IL-17A stimulation was differentially regulated by ERK, JNK, p38 MAPK, and NF-*κ*B pathways [24]. In the present study to investigate the signaling pathways involved in the interaction of basophils/eosinophils and human bronchial epithelial cells upon LIGHT stimulation, several specific signaling molecule inhibitors were used to block the pathways. NF-*κ*B inhibitor BAY11-7082, ERK inhibitor U0126, and p38 MAPK inhibitor SB203580 could differentially suppress LIGHT-induced ICAM-1, IL-6, CXCL8, and MMP-9 in the coculture of basophils/eosinophils and BEAS-2B cells (Figure 7). Together with the results in Figure 6 regarding the LIGHT-mediated activation of ERK, p38 MAPK, and NF-*κ*B in eosinophils alone and BEAS-2B cells in coculture, the results indicated that the induction of ICAM-1 and release of airway remodeling cytokine IL-6, chemokine CXCL8, and extracellular protease MMP-9 in LIGHT-activated coculture of basophils/eosinophils and BEAS-2B cells were differentially regulated by intracellular NF-*κ*B, ERK, and p38 MAPK pathways, probably via the regulation of downstream transcription factors and/or microRNA [22, 50]. These* in vitro* mechanistic results are actually in concordance with our previous published results that the expressions of cytokines/chemokines and adhesion molecules in the coculture of eosinophils/basophils and bronchial epithelial cells are differentially regulated by distinct activation profiles of signaling molecules [22–24]. Since targeting signaling molecules can be a novel strategy for the treatment of asthma [51], the potential cross-talk between different signaling pathways and the downstream molecular regulatory mechanisms awaits further studies.

## 5. Conclusions

In summary, the expression of airway remodeling adhesion molecule ICAM-1 and the release of airway remodeling-related cytokine IL-6, chemokine CXCL8, and extracellular protease MMP-9 and intercellular adhesion were significantly enhanced in the coculture of basophils/eosinophils and bronchial epithelial cells via the regulation of a distinct intracellular signal transduction mechanism. Our previous studies have also shown that the interaction of bronchial epithelial cells and basophils/eosinophils can induce the release of a variety of inflammatory mediators involved in allergic asthma via the upregulated expression of adhesion molecules and the modulation of signaling pathways [22, 23]. The release of mediators such as MMP-9, IL-6, and CXCL8 upon the activation by LIGHT should contribute to airway remodeling. However, whether LIGHT signals through HVEM on basophils/eosinophils and/or LT*β*R on epithelial cells are the direct or indirect consequences involving other receptor-mediated mechanisms requires further investigation. Nevertheless, the present cellular mechanistic results may somehow shed light on the potential therapeutic target for airway remodeling.
